# Immunization against Medically Important Human Coronaviruses of Public Health Concern

**DOI:** 10.1155/2024/9952803

**Published:** 2024-06-08

**Authors:** Nabil A. Nimer, Seema N. Nimer

**Affiliations:** ^1^Faculty of Pharmacy, Philadelphia University, Amman, Jordan; ^2^School of Medicine, The University of Jordan, Amman, Jordan

## Abstract

SARS-CoV-2 is a virus that affects the human immune system. It was observed to be on the rise since the beginning of 2020 and turned into a life-threatening pandemic. Scientists have tried to develop a possible preventive and therapeutic drug against severe acute respiratory syndrome coronavirus-2 (SARS-CoV-2) and other related coronaviruses by assessing COVID-19-recovered persons' immunity. This study aims to review immunization against SARS-CoV-2, along with exploring the interventions that have been developed for the prevention of SARS-CoV-2. This study also highlighted the role of phototherapy in treating SARS-CoV infection. The study adopted a review approach to gathering the information available and the progress that has been made in the treatment and prevention of COVID-19. Various vaccinations, including nucleotide, subunit, and vector-based vaccines, as well as attenuated and inactivated forms that have already been shown to have prophylactic efficacy against the Middle East respiratory syndrome coronavirus (MERS-CoV) and SARS-CoV, have been summarized. Neutralizing and non-neutralizing antibodies are all associated with viral infections. Because there is no specific antiviral vaccine or therapies for coronaviruses, the main treatment strategy is supportive care, which is reinforced by combining broad-spectrum antivirals, convalescent plasma, and corticosteroids. COVID-19 has been a challenge to keep reconsidering the usual approaches to regulatory evaluation as a result of getting mixed and complicated findings on the vaccines, as well as licensing procedures. However, it is observed that medicinal herbs also play an important role in treating infection of the upper respiratory tract, the principal symptom of SARS-CoV due to their natural bioactive composite. However, some Traditional Chinese Medicines contain mutagens and nephrotoxins and the toxicological properties of the majority of Chinese herbal remedies are unknown. Therefore, to treat the COVID-19 infection along with conventional treatment, it is recommended that herb-drug interaction be examined thoroughly.

## 1. Introduction

The atypical form of pneumonia and a new type of coronavirus called novel coronavirus was diagnosed in December 2019 in China, which was referred to as COVID-19 or 2019-nCoV by the World Health Organization. However, the International Committee on Taxonomy of Viruses revised its name to severe acute respiratory syndrome coronavirus-2 (SARS-CoV-2) [1], as there was the identification of the causative agent of this disease in January 2020 as the SARS-CoV-2 virus, with rapid publication of its genome [2]. The symptoms of the disease included difficulty in breathing, affected lungs, fever, and cough [3]. Acute COVID-19 disease is identified as a syndrome of cytokine storms and immunopathogenesis because of the activation of the innate immune system, and these symptoms include tiredness, severe vomiting/nausea, diarrhea, headache, cough, low blood pressure, chest pain and tightness, dyspnea, and high fever and chills. Cytokines perform an important function in regulating in controlling systematic and local inflammatory responses. Proinflammatory cytokines have been reflected as the essential biomarkers for inflammatory and immunology [4]. An increasing impact of COVID-19 was observed by the middle of April 2020 on the global population. This pandemic has affected the whole world causing 6 million deaths. Western Europe has been affected adversely by this virus, with a high prevalence of the affected elderly population, especially the ones with weakened immune systems or having preexisting extensive multiple comorbidities [5], whereas the prevalence of COVID-19 in Asia as of November 2020 reported by WHO was around 10 million. As far as interventions for COVID-19 are concerned, a few points need to be looked upon, which include the need to introduce short-term strategies as well as focused approaches to reduce the effects of COVID-19 among vulnerable populations. Similarly, long-term approaches are also required in parallel to decrease the impact of COVID-19 [2].

The emergence of distinctive numbers of variants and mutations of severe acute respiratory syndrome coronavirus-2 (SARS-CoV-2) has not only affected the lives of the people but also had an impact on several cultural, educational, and socioeconomic activities [6]. The different waves of this epidemic were identified different variants of SARS-CoV-2, including Epsilon (B.1.427/B.1.1429), Theta (P3), Eta (B.1.525), Alpha (B.1.1.7), Beta (B.1.351), Kappa (B.1.617.1), Delta (B.1.617.2), Zeta (P.2), and Gamma (P.1). However, currently, the variant of concern (VOC) B.1.1.529 (Omicron) was emerged from Botswana and South Africa in November 2021 [7]. This Omicron variant has over 30 mutations possibly attacking the S protein. The Omicron variant has the highest rate of transmissibility, as this variant reproduces 70 times faster in human lungs as compared to the Delta variant. The Omicron variant was demonstrated as the most diversified variant. BA.1, BA.2, and BA.3 were the sublineages of the Omicron variant that were accountable for the fourth wave of this pandemic, but BA.4 and BA.5 rapidly emerged as the new sublineage subsequently replaced the former ones [8–11]. The Omicron variant may have arrived from COVID-19-infected patients continuously immunized by non-mRNA or mRNA-based vaccines, permitting the virus to adjust and transform to avoid the immune response. Thus, the effectiveness of already available vaccines produced by using the S protein of former variants of the virus has been debatable. The emerging variants of SAR-CoV-2 have highlighted the significance of doses of booster vaccines to foster defensive immunity in vaccinated people [7, 12, 13]. Therefore, the boosted and vaccinated people have adequate titers of neutralizing antibodies, which are essential for enduring immunity against not only Omicron but also other variants.

SARS-CoV-2 caused a contagious disease that had a destructive effect on public health worldwide. However, the United States was the most affected country globally by COVID-19 as more than 15 million cases of coronavirus and more than 400,000 deaths were reported in the year 2021 [14]. Effective vaccination is demonstrated as the best option to control COVID-19 as it is observed that it has been significantly controlled by using antiviral vaccines, novel diagnostics, and vaccinations. Thus, the study of Liu et al. [15] significantly highlighted the antiviral activity of TRIM56 protein as it has been identified as the host restriction factor. Due to its antiviral barrier function, it significantly plays its role in restricting human coronavirus, HCoV-OC43, and this is directed toward possibilities to target the TRIM56 in developing the antiviral vaccines. However, it is also observed that people are hesitant to vaccinate, and this may be also a potential cause for this endless epidemic. Common attitudes of refusal to get vaccinated against coronavirus have been found in the USA, particularly among females and African Americans. A current survey by Pew Research Centre reported that 39% of adults did not plan to get vaccinated although it is encouraging that this percentage has reduced over time [16, 17].

The management of the strategies against COVID-19 has been a challenge since its outbreak. On the one hand, people are not easy to be convinced to get the vaccination, while on the other hand, the maintenance of vaccination materials is also difficult as it needs a lot of protocols and caution. The maintenance of vaccines requires cold/ultracold storage, and special handling is possible in centralized vaccination clinics like acute healthcare settings based on the implementation of the vaccination program. Given that, there is a need to monitor the safety of vaccines in all the population getting vaccinated under Emergency Use Authorization. Therefore, keeping such situations in consideration, the present study aims to review the available information and progress that has been made for immunization against COVID-19, addressing the potential theoretical safety risks and the emergence of preliminary safety and efficacy data [18].

## 2. Background and Pathogenesis of Coronavirus

**Figure 1:**
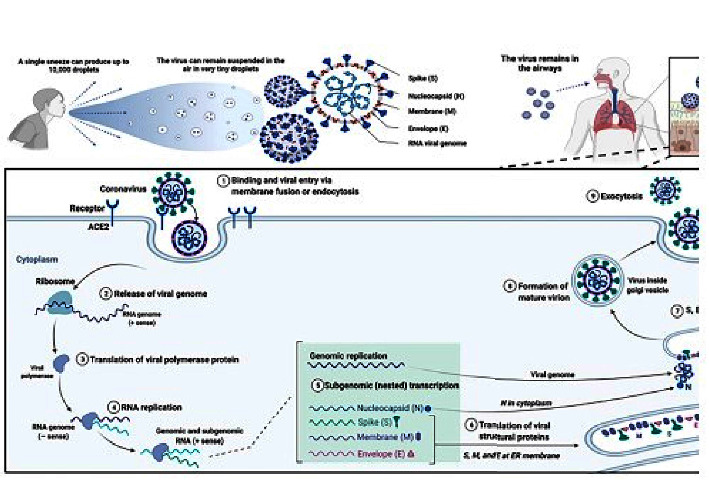
Mechanism of action of SARS-CoV-2 [20].

An unprecedented human pathogen appeared when cough, fever, and dizziness were observed among the workers of seafood markets in the province of China, Hubei, in December 2019. After a couple of weeks, 9976 cases were reported across 21 nations, and within a month, this virus had infected millions of people around the world. However, the pathologic characteristics of COVID-19 vary in severity, acuteness, and distribution. Most patients have the symptoms of the pulmonary and upper respiratory tract, small and large vessel thrombosis, diarrheal symptoms, microvascular injury, cardiac conduction abnormalities, neurologic deficits, and gastrointestinal bleeding may also appear in patients with severe symptoms, this bleeding can be fatal. In other words, the pathogenesis of COVID-19 disease demonstrated that it results from the amalgamation of immune dysregulation and reduction of primary immune cell subsets, interactivity of cascade coagulation, and innate immune system that facilitates inflammatory thromboses [19].  Figure 1 shows the mechanism of action of SARS-CoV-2.

### 2.1. Viral Structure of COVID-19

Coronaviruses infecting birds and mammals are positively sensed, enveloped, and single-stranded RNA viruses [20]. The four structural proteins present in coronavirus are spike (S) proteins, nucleocapsid proteins (N), envelope (E), and membrane (M) proteins that mediate viral entry into cells, forming coronavirus-like particles. The spike protein further consists of S1 and S2 components that are requisite for entry of virus into the cells. The S1 component holds the angiotensin-converting enzyme 2 (ACE2) receptor, while S2 splits transmembrane serine protease-2 (TMPRSS2), thus nurturing the fusion of viruses with the cell membrane.

There is an abundance of angiotensin-converting enzyme 2 on the apical side of alveoli epithelial cells, enterocytes of the small intestine, alveolar monocytes, and macrophages in the lungs, kidneys, and heart involved in the pathogenesis of SARS-CoV [21]. All the antibodies developed against the virus after exposure are not protective. Antibodies developed against the spike protein are considered neutralizing bodies in convalescent serum; however, they do not represent an increased proportion of overall antiviral antibodies [22]. Acute respiratory distress syndrome (ARDS) disseminated intravascular coagulation with fibrin thrombus formation, multiorgan dysfunction, vasculitis, cerebrovascular disease, shock, and even death takes place. ARDS is primarily activated by high levels of proinflammatory cytokines. IL-6 is generally reflected as an imperative proinflammatory cytokine in association with immunopathogenesis of critical COVID-19. Therefore, in handling COVID-19, evaluation of serum level of proinflammatory cytokines can have various implications, such as risk assessment, observation of disease development, prognosis determination, therapy selection, and anticipation of response to treatment. IL-6 has been observed as the inflammatory biomarker frequently related to the progression of COVID-19 disease. Thus, to predict the clinical development of COVID-19, the equilibrium between anti-inflammatory and proinflammatory cytokines may also be beneficial [4]. Hence, it is important to tailor the treatment regime of this disease, appropriately considering its unique features to manage critical and severe cases to limit the mortality rate.

### 2.2. Clinical Significance of COVID Vaccination

Acuteness and severity in COVID-19 disease are featured by cytokine storm because of innate immune activation. Thus, there is a need for acknowledgment and treatment of cytokine storms to deplete the mortality rate in intensified cases of COVID-19 [23] since cytokine storms might result in a bunch of deformities and tissue impairment, which includes malfunctioning of lymphocytes, deformities of granulocytes and monocytes, impairment of vascular barrier, capillary impairment, diffuse alveolar impairment, failure of multiple organs, and even demise [24, 25]. However, patients with primary immunodeficiency (PID) and people with weakened immune systems have a high risk of COVID-19 and also accompany obscured inflammatory responses and cytokine storm [23, 24]. Consecutively, host immune response to infection (SARS-CoV-2) plays an essential part in a clinical demonstration of the immunopathogenesis of COVID-19 patients [4]. Host immune response can be classified into three phases that are elementary innate immune response phase (antiviral defense) in the lungs, the systemic immune response phase (uncontrolled inflammatory response), and the cytokine storm. IL-6 is usually observed as an essential proinflammatory cytokine related to the immunopathogenesis of critical COVID-19 [4, 24]. In some patients of COVID-19, IL-1 and TNF-*α* may also be noticed in high levels. These three cytokines are developed after the stimulation of the innate immune indicating the route. The clinical significance of uncontrolled proinflammatory cytokines has gained much attention. Therefore, Mandel et al. [26] observed that the patients who had a high level of IL-6 and TNF-*α* did not survive and used IL-6 to anticipate 30-day mortality for the COVID-19 patients who were hospitalized with a sensitivity of 97.1%. Furthermore, Chen et al. [19] found detectable serum SARS-CoV-2 viral load and exceptionally high levels of IL-6 in seriously ill patients of COVID-19. In other words, these studies revealed that IL-6 can be considered as a possible therapeutic and medicinal target for critically ill patients with uncontrolled inflammatory responses [4, 24, 25]. Therefore, these studies illustrate the clinical significance of vaccination, which can curtail the length of proinflammatory response followed by SARS-CoV-2 and might reduce the elevated level of cytokines and concentration of chemokine.

### 2.3. Overview of Vaccines

Vaccine development is a time-consuming and complex process that is different from developing conventional medicines. Around 12 to 15 years are required for developing a vaccine [27]. A large number of viruses and bacteria are required in traditional vaccine development methods that last for months, although they are extremely effective to combat highly contagious diseases. Antigens that are the key element in vaccines warn the immune system in humans about the invasion of foreign organisms in the body, which are required to be eliminated [28].

### 2.4. Current Status of COVID-19 Vaccine Development

The importance of precise recognition mechanisms is observable to understand cross-species transmission and host tropism, as well as for the development of animal models vaccines between the host receptors and virus surface proteins [29]. The receptor binding domain is contained in the S1 subunit of the S protein, which is essential for membrane fusion between the virus and the host cell. Different approaches are used to explain the current status of vaccine development against COVID-19.

## 3. Material and Methods

### 3.1. Search Strategy

This study adopted a review approach to gathering the information available and the progress that has been made in the treatment and prevention of COVID-19. Databases such as Google Scholar, PubMed, Web of Science, and Scopus were used to search articles in August 2022 through keywords of SARS-CoV-2/COVID, antibodies, immunization, and vaccines.

### 3.2. Inclusion/Exclusion Criteria

This study included original and review-based studies and excluded reports, and editorials. Moreover, this study only considered articles in English, not any other language. Furthermore, this study included articles that reported information about SAR-CoV-2, their treatment, and vaccination.

## 4. Results

This section discussed on mechanism of action of vaccine candidates and information on various vaccines is presented as follows.

### 4.1. Mechanism of Action of Vaccine Candidates

In addition to the formation of persistent T- and B-cell memory, the most effective approved vaccinations generate long-term antigen-specific antibody responses by plasma cells. SARS-CoV infection needs both humoral and cellular immune responses for infection clearance. By producing axenic target protein in the cytoplasm of the host cell, recombinant viral vectors operate similarly to endogenous pathogens [30]. MHC class 1 molecule delivers such endogenous antigens to CD8+ T-lymphocytes, resulting in the generation of T-cytotoxic cells. This process results in the development of cell-mediated immunity, which is essential for the removal of virus-infected cells.

### 4.2. Vaccination Strategies

Previously, CoV was believed to be a weak virus for humans, causing mild flu-like disease, but recurrent outbreaks, such as SARS in 2002, Middle East respiratory syndrome coronavirus (MERS-CoV) in 2012, and now COVID-19, have proved its pathogenicity internationally [31]. CoV vaccines are highly sought as no antiviral medicines are available against CoV, resulting in worldwide economic losses. Inactivated virus vaccines, recombinant viral vaccines, subunit vaccines, DNA vaccines, and attenuated vaccinations have all been investigated in the hunt for an optimal SARS-CoV vaccine [32].

### 4.3. Nucleic Acid Vaccines

**Figure 2:**
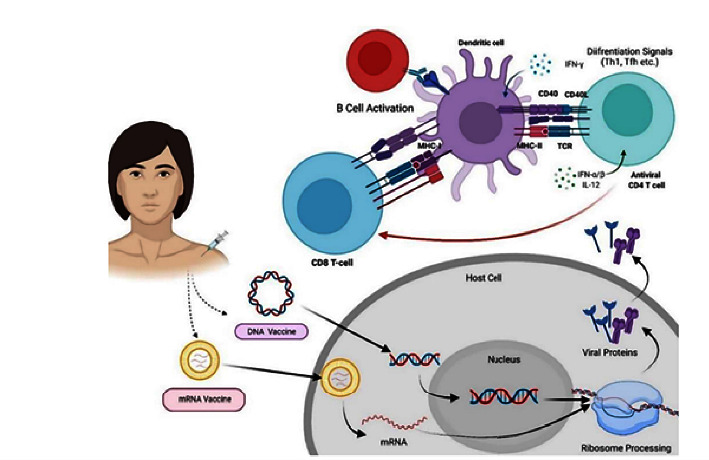
Action of the nucleic acid vaccine [25].

The primary focus of nucleic acid vaccines is on nucleic acid administration to produce RNA or DNA vaccines. The vaccines based on RNA and DNA are unchallenging to create, which permits their immediate production as vaccines. The coding of genomic sequences for specific proteins can be conveniently constructed in DNA or RNA and administered into the cells of the human body to develop numerous copies of viral antigens of immunostimulatory protein. These antigens that are encoded by nucleic acid trigger antibody and cell-mediated responses. Since it facilitates suitable navigation and alteration of the antigens, the technology based on nucleic acid immunization can be regarded as effective and resilient [33]. Furthermore, nucleic acid vaccines can revitalize innate immune responses and antigen-based responses because of their self-adjuvanting characteristics, whereas most kind of vaccines requires adjuvant to perform the parallel objective goal. Once the injection is done, the RNA vaccine does not have access to the nuclear envelope but only enables it to cross the plasma membrane, while DNA vaccines target the nuclear envelope after the encounter of nucleic acid to dendritic cells (DCs), which later develop and offer antigens to T cells to operate resilient immune response. Within DCs, mRNA holds Toll-like receptor 7, whereas DNA holds Toll-like receptor 9 for the operation of Interferon 1 and Th1 cells [34]. Furthermore, the action of the nucleic acid vaccine has been illustrated in Figure 2.

### 4.4. DNA Vaccine

The viral protein with the use of nonreplicating plasmid DNA encoding has a beneficial effect as compared to conventional live attenuated vaccines, as none of the contagious factors is immersed. Since antigenic proteins are endogenously produced and efficiently presented by MHC class I, DNA vaccines consisting of plasmid DNA encoding pathogen-derived proteins induce both T and B cells. It was discovered that antigenic proteins are produced endogenously and presented efficiently by MHC class I, which aids in inducing CD8+ T-cell responses. In addition, DNA vaccines are simple, stable, safe, and easy to manufacture, making them a viable alternative to live vaccines. SARS-CoV proteins, such as S [6, 15, 35], M, and N, have been used as DNA vaccine candidates. SARS-CoV proteins, such as S, M, and N [6, 15, 35], have been reported to produce cellular and humoral responses.

Wolf and Bowers [36] demonstrated naked DNA (N-DNA) or plasmid injected into the muscle cells of the mice. It showed that N-DNA vaccination in mice developed N-specific antibodies and T-cell activity, as well as significant delayed-type hypersensitivity (DTH) and CD8 (+) CTL responses to the N protein. The production of N protein and its connection to LAMP for improved presentation of MHC II has increased memory response in another research of N-DNA vaccination [31]. Furthermore, immunization of C57BL/6 mice with bare CRT/N-DNA for enhanced MHC class I presentation resulted in N-specific humoral and cellular immunity, as well as a significant reduction in the titer of challenging vaccinia virus against N protein-expressing cells [30]. In total 11, DNA-based vaccines for COVID-19 have been structured and are in clinical trials currently on humans. The manufacturers of those vaccines include Inovio Pharmaceuticals, Genexine Consortium, Zydus Cadila, and Symvivo [34].

**Figure 3:**
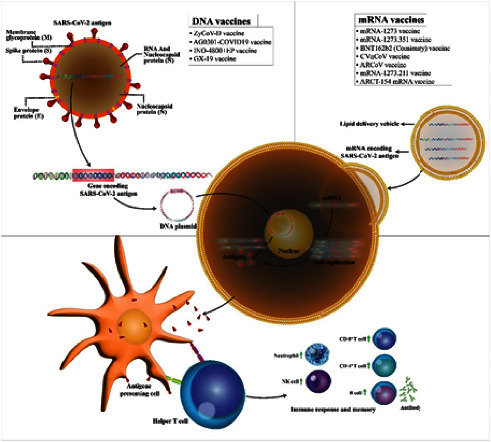
Action of the mechanism of the DNA vaccine candidate [38].

Although DNA vaccines are tolerated by patients and have excellent protective profiles, the most possible adverse effects of DNA vaccines are mild inflammation with pain, irritation, and soreness at the site of vaccination. Furthermore, it was reported that most of the side effects appeared in patients with grade 1/2 (63%, all grade 1) and it was restricted to the site of injection.  Figure 3 shows the action of the mechanism of the DNA vaccine candidate.

### 4.5. mRNA Vaccines

Messenger RNA (mRNA) vaccine is manufactured through RNA vaccine, and this immunization employs a copy of preexistent mRNA to induce an immune response. To pursue an immunization, RNA is transfected into immune cells and transformed into mRNA, which allows the host cells to create extracellular protein. The effective delivery of the mRNA vaccine is possible when it is transmitted into lipid nanoparticles; thus, they attack dendritic cells leading to the inducement of humoral and cellular immune response [34]. The major advantage of the mRNA vaccine is that it eliminates the difficulties involved with the purification of viral proteins, saving months to years in standardization and mass manufacturing [29]. There are currently three authorized RNA vaccines, although clinical trials of 23 vaccines are underway at different stages of development. In cooperation with Moderna, the National Institute of Allergy and Infectious Diseases (NIAID) in the United States has developed an mRNA vaccine called mRNA-1273 [32, 34]. It was found that the SARS-CoV-2 spike protein was produced in a stable state [S]. Furthermore, the administration of RNA vaccines can be done in numerous modalities, which include normal needle injection or needless injection into the epidermis, injection into muscles, blood, and lymph nodes, or direct to the organ or through nasal spray. Although its efficacy is highly demonstrated in clinical trials, it is reported that 58.8% of healthcare workers who are mRNA vaccinated encounter fatigue and 21.99% of vaccinated workers get fever after vaccination [37] (see Figure 3).

### 4.6. Subunit Vaccine

**Figure 4:**
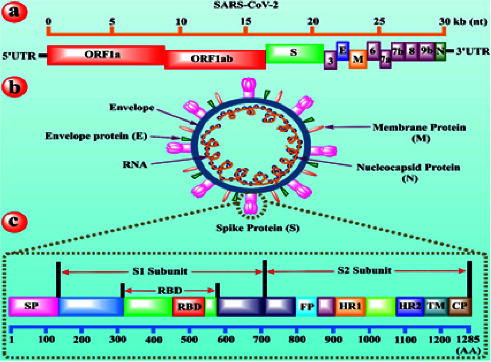
Action of the subunit vaccine candidate [41].

Subunit vaccines are built based on recombinant proteins and synthetic peptides. Contrasting to live attenuated viruses or a few viral vectored vaccines, this kind of vaccine principally encompasses particular fragments of viral antigens but with the exclusion of any constituents of contagious viruses, eradicating distress of partial inactivation, preexistent immunity, or virulence recovery [38]. Subunit vaccinations are less likely to cause adverse effects akin to DNA vaccines at the injection site. Subunit vaccinations are produced reliably due to steady circumstances and specified pathogen pieces. Because of all of these characteristics, subunit vaccines are a promising choice for vaccine development. SARS-CoV produces several structural proteins, including spike (S), envelope (E), membrane (M), and nucleocapsid (N), which may function as antigens to elicit neutralizing antibodies and a defense response [39]. The target of subunit vaccines may be particular, well-identified neutralizing epitopes with enhanced effectiveness or immunogenicity. The side effects of subunit vaccines are mild-to-moderate soreness, fever, and redness at the site of injection.  Figure 4 exhibits the action of the subunit vaccine candidate.

### 4.7. Subunit Vaccines Based on RBD

Subunit vaccines based on RBD are the primary targets in the construction of vaccines for SAR-CoV, as this type of vaccine comprises numerous conformational epitopes having the capacity to generate neutralizing antibodies with high titers. RBD is a 193-AA-residue region in the center of the S1 subunit of the S protein that interacts with receptors in target cells. Antisera from SARS-infected people and animals injected with inactivated SARS-CoV have been shown to successfully react to RBD [40]. RBD eliminated many of the neutralizing antibodies from these antisera by antibody absorption, but these antisera produced antibodies specific to RBD with effective neutralizing activity [41]. Furthermore, RBD generated high-level, long-term SAR-CoV S-particular antibodies and neutralized antibodies that can be conserved for 12 months after immunization and protected vaccinated mice in counter of SAR-CoV infection. RBD protein in a 293T cell expression significantly continues exceptional conformation and excellent antigenicity to bind RBD-specific neutralizing mAbs. Underlining the S protein subunit vaccines, there is no pathogenic impact, which has been found in RBD-based subunit vaccines [42]. This vaccine is very safe, but very common side effects can be experienced after getting vaccinated like tiredness, soreness, fatigue headache, fever, nausea, and vomiting.

### 4.8. Subunit Vaccines Based on S Protein Fragments

The virus's spike protein (S1 and S2) plays an important role in infecting the host since it has manifest protective effectiveness and immunogenicity counter to the infection of SAR-CoV [39]. These types of vaccines are produced by a recombinant synthesis of protein antigens or by the procedure of purifying numerous amounts of pathogen after cultivating. This approach eradicates the probability of extreme effects and increases the requirement to escalate the booster and maximize an added adjuvant to attain strong and long-lasting immunity. The early contacts of the S1 domain with its receptor in the host, PP 4 in MERS-CoV and ACE2 in SARS-CoV, allow the CoV-RNA genome to enter the cells of the host, followed by the viral and host membrane fusion aided by the S2 segment.

### 4.9. Subunit Vaccines Based on M and N Proteins

Apart from the aforementioned proteins, the expression of a variety of additional proteins on the virion surface has been linked to the generation of antibodies in SARS patients' serum. Membrane or M protein is required for maintaining the shape of the viral envelope [43], and it does so by interacting with various CoV proteins, integrating the Golgi complex into new virions [40], and stabilizing nucleocapsid proteins. Studies of animal CoV vaccines show that CoV nucleocapsid protein (N) may be a new target for the virus. However, the N-specified antibodies have been demonstrated in immunized rabbit sera and COVID patients in convalescent phase, but it is found that N-specified antibodies did not have any neutralizing impact against the infection SAR-CoV. Moreover, M protein peptides (M1-31 and M132-161) were used on immunized rabbits and mice sera, and also on SAR patients, it found that its immunogenicity only generated particular IgG antibodies in rabbits. Regardless of their immunogenicity, it seems that subunit vaccines based on N and M proteins have not been explored for their effectiveness in countering the infection of SAR-CoV. Therefore, it is ambiguous if these vaccines can help prevent SAR-CoV infection [6].

### 4.10. Recombinant Vector Vaccines

Recombinant vector vaccines are extremely effective in inducing an immune response because they can infect cells, survive in the body for long periods, and react directly to antigen-presenting cells. In addition, viral proteins in vaccines can function as adjuvants [43], boosting immune response, resulting in the generation of more antibodies and long-term protection, and lowering the antigen dosage necessary. Several attempts to produce a SARS-coronavirus (SARS-CoV) vaccine have been attempted, in which different viral vectors have been genetically modified to express SARS-CoV proteins. Following are the modalities of recombinant vector vaccines.

### 4.11. Adenovirus Vector Vaccine

**Figure 5:**
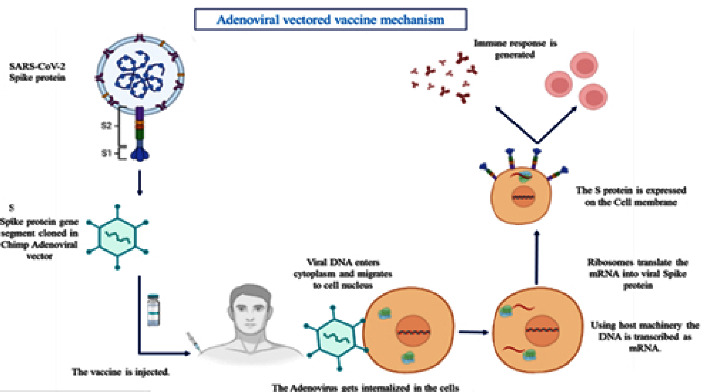
The mechanism of the adenovirus vector.

Adenoviruses are nonenveloped viruses that consist of double-stranded DNA genomic structures. The external part of a virus particle (virion) is a compound of three principal structures of proteins, the fiber, the hexon, and the penton base. Adenovirus-based delivery methods have several advantages, including simplicity of administration via oral or nasal routes, nonpathogenicity in humans, and the ability to target mutants with impaired replication. The adenovirus vector vaccines were first evaluated in mice intranasally as well as intramuscularly [44]. In their study, the vaccine resulted in developing a high level of neutralizing antibodies and reducing RNA in the lungs of the affected mice. It was also found in their study that intranasal delivery of this vaccine is more beneficial in blocking the SARS-CoV in nose and lung tissue. In the same study, they found that the combined adeno-S and adeno-N vaccine resulted in less neutralizing antibodies and similar anti-N IFN-*γ*-secreting cells compared with the inactivated SARS-CoV vaccine. Numerous studies have been conducted on ferrets to assess the efficacy of these vaccines because the ferret is the only animal model that mimics most of the symptoms found in human patients, such as fever, respiratory infections, viral shedding of the upper respiratory tract, and lung injury. When ferrets were primed with an adenoviral construct coding for S protein and boosted with a chimp adenovirus, viral load and other clinical symptoms were significantly reduced, along with being immunogenic in rhesus macaques [45]. Furthermore, the recombinant adenovirus vector (rAd) is stable and easy to manipulate because it can conveniently reduce nonreplication by the removal of the important viral gene E_1_ from its genomic structure [46]. These types of vaccine candidates are established on simian or human adenoviral vectors, and they target generating a defensive immune response of the host by transmitting cells to the pattern of antigenic SDAR-CoV S protein exclusively or in collaboration with the pattern of N protein [47]. On the other hand, adenovirus vector vaccines have severe adverse effects as thrombosis has been reported after the administration of ChAdOx1 nCov-19 vaccination from 1 case per 26000 to 1 case per 127000 doses. It was proposed that vaccination generates platelet-activating antibodies in opposition to platelet factor 4 and becomes a cause of symptoms like autoimmune heparin-induced thrombocytopenia [37]. The mechanism, of the adenovirus vector mechanism, is demonstrated in Figure 5.

### 4.12. Poxvirus Vector Vaccines

Poxvirus vector vaccines are considered to be effective vectors because of their improved stability, larger insert size, manufacturing convenience, cytoplasmic gene expression, and ability to induce long-term cell-mediated and humoral immunity [48]. A study in 2004, delivered the modified vaccinia Ankara (MVA) strain, which is a replication-deficient poxvirus vector encoding the SARS-CoV S protein. Considering the lungs as the target organ in SARS-CoV infection, MVA poxviruses have been accepted as a promising candidate in the construction of COVID-19 vaccines. In this perspective, a unique vaccine program was created for MVA, where a novel three-plasmid structure can effectively produce a chemical synthesis of DNA [49]. The administration led to neutralizing antibodies and reduced viral load in the upper respiratory tract of the affected mice [19]. Similarly, recent studies show the same effectivity of the poxvirus vaccine in hamsters by protecting them from infection [50]. It was found that the double injection of the same vaccine results in a humoral response, including neutralizing antibodies. Awasthi et al. [46] reported that based on two NHP models, it is confirmed that the effectiveness and immunogenicity of TNX-1800, a live virus recombinant poxvirus vaccine candidate, to counter the SAR-CoV infection. The side effects of this vaccine included difficulty in breathing, unusual tiredness, and soreness with the least possibility of allergic reaction [37].

### 4.13. Live Attenuated Vaccines

**Figure 6:**
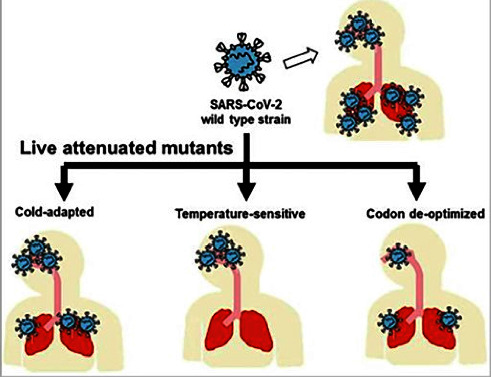
Live attenuated SARS-CoV-2 vaccine candidates [52].

Live attenuated vaccines are manufactured by producing a version of a virus that is genetically weak so that it replicates to a certain limit, not leading to any symptoms of disease but eliciting an immune response that is parallel to natural infection. This vaccine can be provided intranasally; then, it generates a mucosal immune response that provides a defense to the respiratory tract [35]. In addition, the administration of live attenuated SAR-CoV through the nasal tract may encourage the development of IgA, which can prevent infections [37] (see Figure 6). Consequently, in the realm of viral infection, attenuated immunization is a promising technique, but the primary challenge is the pathogen reverting to its virulent form, as happened with the oral poliovirus vaccine [51]. Although live attenuated vaccine is one of the most influential modalities of vaccines, it has some serious problems related to its uses. The problem with the use of live attenuated vaccines is that there is a possibility that viruses can retrieve their harmful substance because of their transformation after vaccination [37]. For such reasons, considering the protection risks, obtaining vaccine regulatory clearance is frequently challenging, and utilizing such vaccinations without solid proof poses a significant risk. Although this limit has not yet been reached for SARS, however, some appealing attenuated mutants have been produced. Moreover, a study found that deletion of the structural E gene resulted in decreased viral morphogenesis and virus titers and consequently weakened the virus [52]. However, other studies do not display similar findings on deletion of genes including ORFs 3a, 3b, 6, 7a, 7b, 8a, 8b, or 9b, showing minimum to no effect on the virus [53]. Therefore, more studies in this direction are required to explore the effect of the deletion of genes in depth.

### 4.14. Risk Factors and Obstacles

Some identified obstacles must be addressed for successful vaccine development collectively with the vaccine technologies. For instance, antibody-dependent enhancement (ADE) of disease is a phenomenon, in which virus entry is enabled via virus-specific antibodies into the host cell through the Fc receptor platform, which leads to aggravated virus infection [43].

There are also host factors of significance, including B- and T-cell responses, particularly the antibody type, class, titer, and the occurrence of compliment. The genetics of the host might further be relevant, which include polymorphism in genes related to adaptive and innate immune responses to viruses including cellular receptor genes, such as Fc gamma receptor (FcgR), genes associated with complement, and cytokine pathways, and histocompatibility complex (MHC) [54].

### 4.15. Existing Approach for Coronaviruses

The main treatment approach for coronaviruses is supportive care, which is supplemented by the unification of broad-spectrum antivirals, convalescent plasma, and corticosteroids (Table 1). The use of HIV protease inhibitors, lopinavir and ritonavir, typically shows adequacy in the treatment of coronaviruses [55]. On the contrary, adverse side effects were revealed for ribavirin including anemia and whether it had immense antiviral activity against coronavirus was unclear. The activity of RNA polymerase was inhibited by nucleoside analogs favipiravir, such as influenza [55–57].

Moreover, COVID-19 might be treated with remdesivir as it possesses in vitro and in vivo antiviral activity against a wide array of RNA viruses, which include MERS and SARS, and can reduce pathology of lungs and viral loads in animal models [58]. The virus-host interactions of coronaviruses might be modulated by host-targeted small molecules approved for other human diseases. For instance, chloroquine had anti-SARS-CoV-2 activity, which is a promising broad-spectrum antiviral drug [59].

Moreover, there is no evidence, indicating that the mortality of MERS and SARS was mitigated by the treatment with corticosteroids. It has been observed that corticosteroid therapy increases clinical features and irregularities in SARS-CoV-affected patients [60]. Thus, corticosteroids are not recommended for systematic use in SARS-CoV-2-infected patients. Additionally, convalescent plasma therapy (CPT) has also been utilized for severe SARS-CoV-2 infection in China and it was indicated that it might have been associated with a few complications like fever, skin erythema, and nausea [61]. However, a few studies reported minimal signs of adverse effects of CPT, with approximately 0.3% mortality rate in the patients [62, 63]. Besides, WHO's recent guidelines on the drugs to prevent COVID-19 affirm the use of hydroxychloroquine, while recommending conditional usage of tixagevimab-cilgavimab in individuals who do not have COVID-19 [64].

Apart from this, the government of China majorly values Traditional Chinese Medicine (TCM) in its campaign for comprising and eradicating previous coronaviruses. For instance, the Health Commission has officially declared that Traditional Chinese Medicine must be utilized instead of traditional medicine in treating COVID-19 patients [65].

### 4.16. Phototherapy: Natural Treatment for COVID

Phototherapy is a natural method of immunization through the practice of medicinal plants in treating and curing coronavirus. Few medicinal plants that hold bioactive composite with CoV-suppressing activities can be employed to treat, prevent, or apply as adjuvant therapies in managing COVID-19. It is interesting to note that 85% of people across Africa, Asia, and the Middle East use herbal medicines as the induction therapy or initial treatment. Furthermore, most people in China extensively use herbs to prevent severe acute respiratory syndrome (SARS) [66]. Some important plants that are used in medicinal therapy are as follows: Andrographis paniculata is a medicinal plant that usually called as King of Bitter. It is extensively used by people in China or tropical Asian regions in treating infectious diseases like upper respiratory tract, inflammation, cold, etc. The main bioactive composite of Andrographis paniculata is andrographolide a diterpenoid that has a broad range of antiviral activities with some other microbial activities. A perennial plant is commonly known as Rhubarb. They are identified to endeavor some antiviral activities through emodin to control CoV. In addition, it was advocated that emodin is regarded as a possible principal therapeutic factor for SAR. Polygonum multiflorum Thunb is also called tuber fleece flower. It is Chinese medicine registered in the list of Chinese Pharmacopoeia. After the laboratory testing, it was found that Polygonum multiflorum has different bioactive composite with antiviral, inflammatory, antioxidant, and antibacterial characteristics. The most important bioactive composite of this plant is emodin similar to Rhubarb. The common name of Glycyrrhiza glabra is liquorice. This plant is broadly available in Europe and Asia. It has glycyrrhizin as the important bioactive composite, which serves as an obstructing interaction between ACE2 and S protein, and thus, it provides help in the prevention of viral invasion [66].

Undoubtedly, nature is the best way to treat infections and other diseases. It has an advantage over medicines since it is easily available and accessible as well and they are less harmful in comparison with drug synthesis. Zhang and Liu [37] reported that treatment of SAR-CoV through medicinal plants provided some comfort on the verge of a pandemic. Due to the natural bioactive composite, plants play an important role in treating infection of the upper respiratory tract, the principal symptom of SAR-CoV. Different vaccines, including nucleotide-based, subunit, vector-based, attenuated, and inactivated, show their effectiveness in preventing MERS-CoV and SARS-CoV infections [67]. Therefore, they could serve as valuable options for creating a safe and effective vaccine against SARS-CoV-2.

## 5. Conclusions

Since the outbreak of COVID-19, there has been an instant pace toward vaccine development. However, COVID-19 has triggered and has been a challenge to keep reconsidering the usual approaches to regulatory evaluation as a result of getting mixed and complicated findings on the vaccines, as well as licensing procedures. Vaccine companies are indicating a preference for scaling up production before reaching a definitive phase. Therefore, the integration of high quality is important to assess real-world effectiveness. Of a new vaccine against COVID-19, we aligned with its surveillance across multiple regions and demographics. This also highlights the phototherapy to cure and treat SAR-CoV infection naturally. We found that plant-based medicines are more easily available and accessible to every corner of the globe and they are also affordable as compared to synthesized drugs. We also discussed some medicinal plants that have a different bioactive composite with antioxidant, antiviral, and antibacterial characteristics that could help prevent contagious infections and diseases.

This study did not review the safety of Traditional Chinese Medicine in the treatment of a recent COVID-19 infection. Some Traditional Chinese Medicine herbs include mutagens and nephrotoxins; however, the toxicological properties of the majority of Chinese herbal remedies are unknown. However, the perennial prevalence of the adversarial effect of Traditional Chinese Medicine limits its internalization and modification. Therefore, the safety of Traditional Chinese Medicine when used to treat COVID-19 infection along with the efficacy of conventional treatment due to herb-drug interactions can be thoroughly examined.

### 5.1. Limitation of Study

This study has limitations as this study is based on a review approach. In the future, researchers should focus on the quantitative and qualitative approach to identify the after-effects of vaccinations on vaccinated people through interviews and survey-based studies as the COVID-19 epidemic is promptly progressing incidence and extensive research on immunization against SARS-CoV-2 will accumulate and grow rapidly.

## Figures and Tables

**Table 1:** Traditional treatment of patients with COVID-19.

Type of treatment	Therapeutic agent or device
Oxygen therapy	Nasal cannula
	Noninvasive mechanical ventilation
	Invasive mechanical ventilation
	ECMO

Anti-inflammatory therapy	Abatacept
	Infliximab

Antivirals	Lopinavir/ritonavir
	Ribavirin
	Favipiravir (T-705)
	Remdesivir
	Oseltamivir
	Chloroquine

*Note*. ECMO: extracorporeal membrane oxygenation.

## Data Availability

The data will be available for review from the corresponding author upon request.

## Additional Points

*Permission Statement for Figures*. The figures have been adopted and substantiated from the published materials that have allowed redistribution of their materials for educational and academic purposes as they are published under the Creative Commons license.

## References

[1] Coronaviridae Study Group of the International Committee on Taxonomy of Viruses (2020). The species Severe acute respiratory syndrome-related coronavirus: classifying 2019-nCoV and naming it SARS-CoV-2. *Nature Microbiology*.

[2] Yuen K. S., Ye Z. W., Fung S. Y., Chan C. P., Jin D. Y. (2020). SARS-CoV-2 and COVID-19: the most important research questions. *Cell and Bioscience*.

[3] Song F., Shi N., Shan F. (2020). Emerging 2019 novel coronavirus (2019-nCoV) pneumonia. *Radiology*.

[4] Liu B. M., Martins T. B., Peterson L. K., Hill H. R. (2021). Clinical significance of measuring serum cytokine levels as inflammatory biomarkers in adult and pediatric COVID-19 cases: a review. *Cytokine*.

[5] Wyper G. M., Assunção R., Cuschieri S. (2020). Population vulnerability to COVID-19 in Europe: a burden of disease analysis. *Archives of Public Health*.

[6] Mohapatra R. K., Kuppili S., Kumar Suvvari T. (2022). SARS‐CoV‐2 and its variants of concern including Omicron: a never ending pandemic. *Chemical Biology and Drug Design*.

[7] Mohapatra R. K., El-Shall N. A., Tiwari R. (2022). Need of booster vaccine doses to counteract the emergence of SARS-CoV-2 variants in the context of the Omicron variant and increasing COVID-19 cases: an update. *Human Vaccines and Immunotherapeutics*.

[8] Mohapatra R. K., Mahal A., Kutikuppala L. S. (2022). Renewed global threat by the novel SARS-CoV-2 variants ‘XBB, BF. 7, BQ. 1, BA. 2.75, BA. 4.6’: a discussion. *Frontiers in Virology*.

[9] Mohapatra R. K., Verma S., Kandi V. (2023). The SARS‐CoV‐2 Omicron variant and its multiple sub‐lineages: transmissibility, vaccine development, antiviral drugs, monoclonal antibodies, and strategies for infection control–a review. *ChemistrySelect*.

[10] Mohapatra R. K., Mishra S., Kandi V. (2023). Analyzing the emerging patterns of SARS‐CoV‐2 Omicron subvariants for the development of next‐gen vaccine: an observational study. *Health Science Reports*.

[11] Mohapatra R. K., Mahal A., Mishra S., Kandi V., Obaidullah W. J. (2023). SARS-CoV-2 variants BA. 2.86 and EG. 5.1 alongside scrub typhus and nipah in India during the ongoing cricket world cup 2023: threat perceptions and countermeasures. *Cureus*.

[12] Wahid M., Jawed A., Mandal R. K. (2023). Role of available COVID-19 vaccines in reducing deaths and perspective for next generation vaccines and therapies to counter emerging viral variants: an update. *Minerva Medica*.

[13] Satapathy B. S., Pattnaik G., Sahoo R. N. (2023). COVID‐19 vaccines and their underbelly: are we going the right way?. *Health Science Reports*.

[14] Momplaisir F., Haynes N., Nkwihoreze H., Nelson M., Werner R. M., Jemmott J. (2021). Understanding drivers of coronavirus disease 2019 vaccine hesitancy among blacks. *Clinical Infectious Diseases*.

[15] Liu B., Li N. L., Wang J. (2014). Overlapping and distinct molecular determinants dictating the antiviral activities of TRIM56 against flaviviruses and coronavirus. *Journal of Virology*.

[16] Fisk R. J. (2021). Barriers to vaccination for coronavirus disease 2019 (COVID-19) control: experience from the United States. *Global Health Journal*.

[17] Allington D., McAndrew S., Moxham-Hall V., Duffy B. (2023). Coronavirus conspiracy suspicions, general vaccine attitudes, trust and coronavirus information source as predictors of vaccine hesitancy among UK residents during the COVID-19 pandemic. *Psychological Medicine*.

[18] Bennet B. M., Wolf J., Laureano R., Sellers R. S. (2020). Review of current vaccine development strategies to prevent coronavirus disease 2019 (COVID-19). *Toxicologic Pathology*.

[19] Chen X., Zhao B., Qu Y. (2020). Detectable serum SARS-CoV-2 viral load (RNAaemia) is closely associated with drastically elevated interleukin 6 (IL-6) level in critically ill COVID-19 patients. *medRxiv*.

[20] Lurie N., Sharfstein J. M., Goodman J. L. (2020). The development of COVID-19 vaccines: safeguards needed. *JAMA*.

[21] McClung N., Chamberland M., Kinlaw K. (2020). The Advisory Committee on Immunization Practices’ ethical principles for allocating initial supplies of COVID-19 vaccine—United States, 2020. *MMWR Morbidity and mortality weekly report*.

[22] Schoeman D., Fielding B. C. (2019). Coronavirus envelope protein: current knowledge. *Virology Journal*.

[23] Chavda V. P., Hossain M. K., Beladiya J., Apostolopoulos V. (2021). Nucleic acid vaccines for COVID-19: a paradigm shift in the vaccine development arena. *Biologics*.

[24] Liu B. M., Hill H. R. (2020). Role of host immune and inflammatory responses in COVID-19 cases with underlying primary immunodeficiency: a review. *Journal of Interferon and Cytokine Research*.

[25] Wang N., Shang J., Jiang S., Du L. (2020). Subunit vaccines against emerging pathogenic human coronaviruses. *Frontiers in Microbiology*.

[26] Mandel M., Harari G., Gurevich M., Achiron A. (2020). Cytokine prediction of mortality in COVID19 patients. *Cytokine*.

[27] Robbiani D. F., Gaebler C., Muecksch F. (2020). Convergent antibody responses to SARS-CoV-2 in convalescent individuals. *Nature*.

[28] Zhang C., Maruggi G., Shan H., Li J. (2019). Advances in mRNA vaccines for infectious diseases. *Frontiers in Immunology*.

[29] Guarner J. (2020). Three emerging coronaviruses in two decades: the story of SARS, MERS, and now COVID-19. *American Journal of Clinical Pathology*.

[30] Du L., He Y., Zhou Y., Liu S., Zheng B. J., Jiang S. (2009). The spike protein of SARS-CoV—a target for vaccine and therapeutic development. *Nature Reviews Microbiology*.

[31] Sun C., Chen L., Yang J. (2020). SARS-CoV-2 and SARS-CoV spike-RBD structure and receptor binding comparison and potential implications on neutralizing antibody and vaccine development. *bioRxiv*.

[32] Chan J. F., Lau S. K., To K. K., Cheng V. C. C., Woo P. C. Y., Yuen K. Y. (2015). Middle East respiratory syndrome coronavirus: another zoonotic betacoronavirus causing SARS-like disease. *Clinical Microbiology Reviews*.

[33] Vogel F. R., Sarver N. (1995). Nucleic acid vaccines. *Clinical Microbiology Reviews*.

[34] Coughlan L. (2020). Factors which contribute to the immunogenicity of non-replicating adenoviral vectored vaccines. *Frontiers in Immunology*.

[35] Alagheband Bahrami A., Azargoonjahromi A., Sadraei S., Aarabi A., Payandeh Z., Rajabibazl M. (2022). An overview of current drugs and prophylactic vaccines for coronavirus disease 2019 (COVID-19). *Cellular and Molecular Biology Letters*.

[36] Wolf M., Bowers P. G. (1999). The double-deficit hypothesis for the developmental dyslexias. *Journal of Educational Psychology*.

[37] Zhang L., Liu Y. (2020). Potential interventions for novel coronavirus in China: a systematic review. *Journal of Medical Virology*.

[38] Baldo A., Leunda A., Willemarck N., Pauwels K. (2021). Environmental risk assessment of recombinant viral vector vaccines against SARS-Cov-2. *Vaccines*.

[39] Zakhartchouk A. N., Viswanathan S., Moshynskyy I., Petric M., Babiuk L. A. (2007). Optimization of a DNA vaccine against SARS. *DNA and Cell Biology*.

[40] Kim T. W., Lee J. H., Hung C. F. (2004). Generation and characterization of DNA vaccines targeting the nucleocapsid protein of severe acute respiratory syndrome coronavirus. *Journal of Virology*.

[41] Pardi N., Hogan M. J., Porter F. W., Weissman D. (2018). mRNA vaccines—a new era in vaccinology. *Nature Reviews Drug Discovery*.

[42] Lundstrom K. (2020). Application of viral vectors for vaccine development with a special emphasis on COVID-19. *Viruses*.

[43] Li F. (2016). Structure, function, and evolution of coronavirus spike proteins. *Annual review of virology*.

[44] See R. H., Zakhartchouk A. N., Petric M. (2006). Comparative evaluation of two severe acute respiratory syndrome (SARS) vaccine candidates in mice challenged with SARS coronavirus. *Journal of General Virology*.

[45] Bisht H., Roberts A., Vogel L. (2004). Severe acute respiratory syndrome coronavirus spike protein expressed by attenuated vaccinia virus protectively immunizes mice. *Proceedings of the National Academy of Sciences*.

[46] Awasthi M., Macaluso A., Myscofski D. (2023). Immunogenicity and efficacy of TNX-1800, A live virus recombinant poxvirus vaccine candidate, against SARS-CoV-2 challenge in nonhuman primates. *Vaccines*.

[47] Krammer F. (2020). SARS-CoV-2 vaccines in development. *Nature*.

[48] Rocha C. D., Caetano B. C., Machado A. V., Bruña-Romero O. (2004). Recombinant viruses as tools to induce protective cellular immunity against infectious diseases. *International Microbiology: The Official Journal of the Spanish Society for Microbiology*.

[49] Okamura S., Ebina H. (2021). Could live attenuated vaccines better control COVID-19?. *Vaccine*.

[50] Harrop R., Connolly N., Redchenko I. (2006). Vaccination of colorectal cancer patients with modified vaccinia Ankara delivering the tumor antigen 5T4 (TroVax) induces immune responses which correlate with disease control: a phase I/II trial. *Clinical Cancer Research*.

[51] Roper R. L., Rehm K. E. (2009). SARS vaccines: where are we?. *Expert Review of Vaccines*.

[52] DeDiego M. L., Álvarez E., Almazán F. (2007). A severe acute respiratory syndrome coronavirus that lacks the E gene is attenuated in vitro and in vivo. *Journal of Virology*.

[53] DeDiego M. L., Pewe L., Alvarez E., Rejas M. T., Perlman S., Enjuanes L. (2008). Pathogenicity of severe acute respiratory coronavirus deletion mutants in hACE-2 transgenic mice. *Virology*.

[54] Zhang J., Zeng H., Gu J., Li H., Zheng L., Zou Q. (2020). Progress and prospects on vaccine development against SARS-CoV-2. *Vaccines*.

[55] Smatti M. K., Al Thani A. A., Yassine H. M. (2018). Viral-induced enhanced disease illness. *Frontiers in Microbiology*.

[56] Duffy S. (2018). Why are RNA virus mutation rates so damn high?. *PLoS Biology*.

[57] Borczuk A. C., Yantiss R. K. (2022). The pathogenesis of coronavirus-19 disease. *Journal of Biomedical Science*.

[58] De Clercq E. (2019). New nucleoside analogues for the treatment of hemorrhagic fever virus infections. *Chemistry--An Asian Journal*.

[59] Holshue M. L., DeBolt C., Lindquist S. (2020). First case of 2019 novel coronavirus in the United States. *New England Journal of Medicine*.

[60] Wu J., Huang J., Zhu G. (2020). Systemic corticosteroids and mortality in severe and critical COVID-19 patients in Wuhan, China. *Journal of Clinical Endocrinology and Metabolism*.

[61] Duan K., Liu B., Li C. (2020). Effectiveness of convalescent plasma therapy in severe COVID-19 patients. *Proceedings of the National Academy of Sciences*.

[62] Huang C., Wang Y., Li X. (2020). Clinical features of patients infected with 2019 novel coronavirus in Wuhan, China. *The Lancet*.

[63] Joyner M. J., Wright R. S., Fairweather D. (2020). Early safety indicators of COVID-19 convalescent plasma in 5000 patients. *Journal of Clinical Investigation*.

[64] World Health Organization (2023). *Drugs to Prevent COVID-19: Living Guideline*.

[65] National Health Commission of the People’s Republic of China (2020). http://www.nhc.gov.cn/xcs/s3574/202002/f12a62d10c2a48c6895cedf2faea6e1f.shtml.

[66] Adeleye O. A., Bamiro O. A., Bakre L. G. (2022). Medicinal plants with potential inhibitory bioactive compounds against coronaviruses. *Advanced Pharmaceutical Bulletin*.

[67] Pandey S. C., Pande V., Sati D., Upreti S., Samant M. (2020). Vaccination strategies to combat novel corona virus SARS-CoV-2. *Life Sciences*.

